# Current Status and Future Potential of Machine Learning in Diagnostic Imaging of Endometriosis : A Literature Review

**DOI:** 10.31729/jnma.8897

**Published:** 2025-03-31

**Authors:** Palpasa Shrestha, Bibek Shrestha, Jati Sherestha, Jun Chen

**Affiliations:** 1 Department of Radiology, Kenmin Hospital of Wuhan University, Jiefang Koad, Wuhan, Hubei Province, People's Republic of China; 2 Department of Kadiology, Zhongnan Hospital of Wuhan University, Wuhan, Hubei Province People's Republic of China; 3 National Trauma Center, Mahakal, Kathamandu, Nepal

**Keywords:** *artificial intelligence*, *diagnostic imaging*, *endometriosis*, *machine learning*

## Abstract

The presence of endometrial tissue outside the uterus is a defining characteristic of endometriosis, a chronic systemic illness that affects women of childbearing age. Despite its enigmatic nature, laparoscopy remains the gold standard for diagnosis, while noninvasive methods such as transvaginal ultrasonography and magnetic resonance imaging are commonly used to aid in preoperative planning. In healthcare, AI has emerged as a game-changing innovation, enhancing patient outcomes, reducing costs, and revolutionizing healthcare delivery, particularly in diagnostic radiology. Images can be analyzed using machine learning, a pattern recognition method. The machine learning algorithm first computes the image characteristics deemed significant for making predictions or diagnoses about unseen images.

## INTRODUCTION

The utilization of AI in medicine is on the rise, demonstrating significant potential in diagnosis, treatment, and data analysis^1,2^. AI in endometriosis diagnosis has been explored in various contexts, including the recognition of ovarian endometriosis cancer^3^, the application of MRI ^4^, and the classification of laparoscopic images.^5^ Within the AI, ML encompasses techniques such as logistic regression that utilize both training and test data.^2^ Studies show ML models achieve high accuracy (AUC 0.5-0.9) in predicting endometriosis.^6,7^ It also analyzes comorbidities, genetic data, and imaging to understand endometriosis comprehensively.^4,8^ The research landscape highlights gaps for exploration, as endometriosis is associated with conditions such as ovarian cancer and multimorbidity. However, additional research is essential to comprehend the mechanisms connecting endometriosis with these comorbidities and to create ML models for predicting and managing the disease complexities.^9^ Integrating ML with emerging technologies could enhance our understanding of endometriosis's genetic and molecular foundations.^10^

## METHODOLOGY

This review employs a systematic method involving a thorough search across various databases (PubMed, Scopus, Google Scholar) for studies published between [2010] and [2024], using mesh terms “machine learning”, “diagnostic imaging”, “endometriosis”, “artificial intelligence”, and “deep learning”. Although no language restriction were imposed, only studies published in english were eligible for inclusion.

**Table:** 

Inclusion Criteria	Exclusion Criteria
Research focused on the application of artiticial intelligence or machine learning to endometriosis diagnostic imaging.	Research on imaging techniques (such as non-abdominal imaging) that are not relevant to endometriosis.
Research examining the diagnosis, lesion identification, and endometriosis staging using ML approaches such as Deep learning, support vector machines, and neural networks.	Studies with insufficient methodological rigor or those that doesn't produce thorough findings.
Reviews, Randomized research, or clinical trials evaluating the performance of machine learning models in real or simulated clinical situations.	Non English language
Studies published in English Language	

## CURRENT DIAGNOSTIC IMAGING

Laparoscopy is limited in diagnosing endometriosis due to its invasiveness, risk, and cost. Ultrasonography(USG) and MRI are noninvasive diagnostic methods for endometriosis.^11^ Deep learning excels in bioinformatics, including mutation map analysis and ML models for diagnosing endometriosis using transcriptomics and methylomics data.^12^ Recent studies show that multi-scale deep learning ensembles can enhance the segmentation and detection of endometriotic lesions from TVUS and MRI images.^13^ AI identifies patterns in data and analyzes large datasets, aiding endometriosis research for developing diagnostic ultrasound tools and predicting treatment success.^14^ A scoping review examined AI methods for clinical issues in endometriosis, covering pathology, diagnostics, prediction, and management, along with evaluation metrics for validated models.^15^

## ULTRASONOGRAPHY IN DIAGNOSTIC IMAGING

The exactness of TVUS in the diagnosis of endometriosis depends upon the location of the lesions, and it is best used to detect ovarian and rectal deep endometriosis, where limitations are poor in the detection of uterosacral ligament deep endometriosis, bladder deep endometriosis and vaginal deep endometriosis.^16^

**Ovarian Endometriosis:** In premenopausal and postmenopausal women, the disorder is characterized by a "unilocular cyst with ground glass echogenicity of the cyst fluid," which may fluctuate. Endometriosis, which can cause papillate and endometriomas, is more common in the elderly than in younger women. In diagnosing endometriomas using TVUS, the sensitivity is 91%, and the specificity is 96%.^17^

**Superficial endometriosis:** Thicker pericolic fat is an indication of superficial endometriosis. TVUS has relatively low sensitivity and specificity for diagnosing superficial endometriosis.^17^

**Deep Endometriosis (DE):** DE is usually diagnosed using ultrasonography using the International Deep Endometriosis Analysis (IDEA) protocol. Four phases in the IDEA protocol must be performed when performing TVUS on a patient who may have endometriosis.^18^

**Anterior Endometriosis:** Anterior compartment DE includes hypoechoic lesions involving the muscularis, mucosa of the bladder or the uterovesical space. If the posterior bladder moves freely over the anterior uterine wall, the uterovesical space is non obliterated. The sensitivity and specificity for detecting bladder endometriosis are 62% and 100%, respectively. The sensitivity and specificity for detecting endometriosis in the rectovaginal space were 49% and 98%, respectively. For diagnosing ureteral endometriosis, the sensitivity and specificity are 92% and 100%, respectively.^17^

Posterior and lateral endometriosis: Uterosacral ligament endometriosis presents as nodules with regular or irregular margins and hyperechoic points and has a sensitivity of 53% and a specificity of 93%. Vaginal endometriosis, which is suspected when the posterior vaginal fornix is thickened or if there is a hypoechoic nodule, has a sensitivity of 52% and a specificity of 98%. In a rectosigmoid colon, an endometriotic lesion typically manifests as an irregular hypoechoic nodule within the wall, exhibiting a reported sensitivity of 91% and a specificity of 97%. TVUS also aids in measuring the distance from the lesion to the anal verge in rectosigmoid DE.^17^

With the use of ultrasound markers such as ovarian immobility and endometriomas, transvaginal sonography, a "sliding sign”, aids in the evaluation of disease severity and is associated with POD obliteration and posterior compartment DIE.^19^

## MRI IN DIAGNOSTIC IMAGING

The most common MR protocols for endometriosis are T2-weighted imaging (T2WI) (axial, coronal, and sagittal) and T1-weighted imaging (T1WI) with fat saturation (axial and sagittal). Contrast and diffusion-weighted imaging (DWI) can sometimes be used. T1-fat saturation (T1-fatsat) helps distinguish between endometriomas and mature cystic teratomas, which typically contain fat.

The peritoneum, ovaries, and uterine ligaments are all covered in superficial plaques in superficial endometriosis. These lesions appear to be microcystic and micronodular. Many round (cystic or nodular) lesions that are homogeneously hyperintense on fat saturation T1WI appear due to hemorrhagic material.^20^

**Figure 1 a, b, c:**
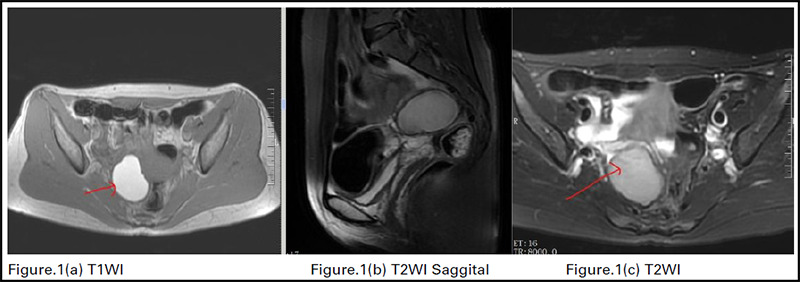
Shows Short T2 signal (hypointense) in the right ovary, considering the possibility of endometriosis and chocolate cyst

Endometriomas exhibit increased signal intensity on T1WI and T1-Fatsat sequences. On T2WI, they can appear to have a lower signal intensity, also known as shading, or they may have an intermediate intensity. This can be caused by blood products from multiple bleeding events in different stages of decay. These follicles are compared to typical nearby ovarian follicles.^20^ The "T2 dark spot sign" is a more precise indicator of ovarian endometriomas. It is defined as hypointense foci within the cyst on T2WI, with or without T2 Shading, also known as chocolate cyst (Figure 1a, b, c).^21^ Adhesion between adjacent peritoneal surfaces can cause the ovaries to merge in the pouch of Douglas; this process is referred to as "kissing the ovaries" and is diagnostic of severe pelvic endometriosis.^20^

Deep endometriosis is characterized by implants or tissue masses ^22^, and lesions in the retro cervical area usually appear on T2WI images as poorly defined infiltrative tissue that is hypointense and extends from the posterior uterine serosa to the retro cervical region.^23^ On T1WI, these lesions consistently exhibit low to intermediate signal intensity. Intestinal endometriosis manifests as a thicker wall with low signal T2 and iso signal T1 intensity, correlating to fibrosis and muscle hypertrophy, with or without cystic or hemorrhagic foci detectable on T2WI or T1WI.^24^

## ENHANCED DIAGNOSTIC IMAGING POWDERED BY MACHINE LEARNINGS

Machine learning, a specialized branch of artificial intelligence(AI), was first defined by the esteemed computer scientist Arthur Samuel in 1959 ^25^. This technology enables the detection of meaningful patterns within various datasets. When a machine learning algorithm is employed to analyze a patient's dataset, including age, sex, family history, imaging results, and relevant contextual information, the algorithm can extract knowledge from the data. Subsequently, it can apply this acquired knowledge to predict outcomes in novel, unseen datasets. This predictive capability enhances the accuracy of the forecasts and significantly enriches the insights gained from the analysis, ultimately leading to improved clinical decision-making and patient care. As such, this application of machine learning in healthcare represents a transformative advancement in data utilization for enhancing medical outcomes ^26^.

There are different types of machine learning based on the needs of data. Supervised learning, unsupervised learning, semi-supervised learning, reinforcement learning, transduction, and learning to learn ^27^. Supervised machine learning is developed for specific finite data that is determined by a human, and in practice, a particular segment of data will be labeled with a specific classification. The task of machine learning is to find a pattern and construct a model where these models are then evaluated based on their predictive capacity about the measure of variance in the data itself ^27^.

Radiological imaging uses supervised machine learning, where a database of radiological images is used to train an algorithm that produces specific classification output ^28,29^. Once the algorithms are thoroughly trained, they can be leveraged to classify or predict previously unseen test images with a high degree of accuracy ^30^. Recent advancements in artificial intelligence, machine learning, and deep learning are rapidly emerging as transformative, data-driven methodologies for addressing various complex medical challenges, including endometriosis ^31,32^. The integration of AI in the domain of endometriosis can be categorized into three primary areas: the development of diagnostic models, the prediction of population-level outcomes, and the optimization of research efficiency ^33^.

**Figure 2:**
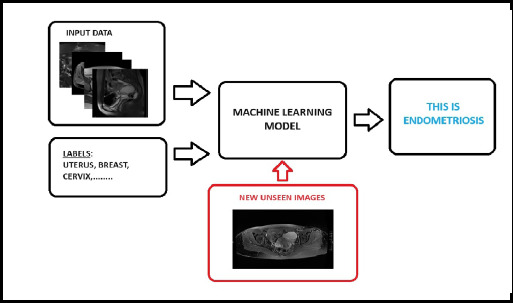
Flowchart of supervised machine learning showing mechanism of how ML works

The benefits of machine learning in the medical field can be outlined as follows:

AI and ML-assisted detection and diagnosis can support physicians in interpreting medical imaging results, significantly reducing interpretation time. These algorithms have been applied across various modalities, including CT, MRI, and Mammography ^34^. Supervised machine learning involves acquiring knowledge through analyzing endometriosis images alongside critical patient data such as age, risk factors, and family history. This learned information is then applied to the presence of endometriosis in previously unseen pelvic images. In this process, the algorithm is trained on a dataset containing labeled images of severe endometriosis annotated with patient age and associated risk factors. Subsequently, the model can determine whether a given lesion corresponds to endometriosis (Figure 2).

There are various supervised machine learning algorithms including Decision Tree, K-nearest neighbor, Random Forest, Linear regression, Support vector, Adaptive Boost (Ada Boost), Neural Network Method, Logistic regression, Naive bayes^35^ used in field of medicine. Among deep learning algorithms, Convolutional Neural Networks (CNNs) are the most widely utilized in radiology, demonstrating high efficiency in tasks such as image segmentation and classification.^36^ CNNs are often called neural networks due to their structural similarity to human brain neurons. For instance, lower levels of input information are connected to the subsequent layer, which then synthesizes this data to produce a more intricate and complex output.^34^ Simple neural network-based machine learning algorithms generally comprise a limited number of layers, whereas deep learning algorithms can incorporate significantly more layers than traditional machine learning models. The inclusion of additional layers has been demonstrated to enhance test accuracy.^37^

There are two primary categories of deep learning applications in reporting: image reconstruction and workstation post-processing.^38^ Numerous studies have demonstrated a substantial reduction in reconstruction time when employing deep learning-based reconstruction methodologies.^39,40^ Turbo spin-echo (TSE) imaging is extensively utilized in musculoskeletal and pelvic imaging because of its robustness and top-notch image quality. This approach provides a remarkable 65% reduction in reconstruction time without compromising image quality.^39^ The rising volume of radiological examinations and the increasing number of images acquired for computer-aided analysis have proven invaluable in daily clinical practice. Deep learning networks, particularly CNNs, are ideally suited for advanced image data analysis, facilitating the detection of specific structures and findings.^38^

The CNNs algorithm successfully detected the presence of endometriosis on ultrasound with an area under curve (AUC) of 90% and an accuracy of 80%. This algorithm has been very useful in medicine because it can be used for patient ultrasounds. It is suggested that 90% probability and 80% accuracy be used to determine whether the patient has endometriosis or not.^41^ An artificial neuron network (ANN) model of deep learning has shown a sensitivity and specificity of 72% and 73%, respectively, for endometriosis.^42^ The fuzzy C-means (FCM) algorithm applied to MRI images used for segmentation achieved an accuracy of 94.32% ± 3.05%, much greater than that of traditional MRI 81.39%± 3.11%.^43^

## LIMITATIONS OF MACHINE LEARNING APPROACHES

One significant limitation of these machine learning methods is their tendency to overfit, particularly with small datasets.^44^ To tackle this, researchers stress the importance of extensive, well-organized datasets for training strong and versatile models.^6-8^ Moreover, employing machine learning methods designed for longitudinal data, like recurrent neural networks (RNNs) or survival analysis models, could effectively capture endometriosis's intricate and varied trajectory throughout a woman's life.^45^ To overcome these challenges, a comprehensive strategy incorporating machine learning, imaging, and additional diagnostic methods (such as biomarkers and genetic information) may be needed to enhance the accuracy and dependability of endometriosis diagnosis.^3,15^ More research is necessary to create reliable risk models for predicting endometriosis-associated ovarian cancer (EAOC) in endometriosis patients.^3^

## CHALLENGES AND FUTURE DIRECTIONS

While ML approaches show great promise, several challenges must be addressed for effective implementation. The quality and quantity of annotated training data are essential for building robust models, and external validation across diverse populations is needed to ensure generalizability. Integrating ML models into clinical workflows requires a multidisciplinary approach involving radiologists, gynecologists, and data scientists. Variability in imaging protocols, equipment, and patient factors can lead to inconsistent feature extraction, highlighting the need for standardized protocols to enhance data quality.

A considerable amount of research remains to be conducted regarding the application of artificial intelligence in studying endometriosis. Given the current limitations in resources and the relatively sparse body of existing studies, further investigations must be undertaken to fully explore the potential of AI technologies in enhancing our understanding and management of this complex condition. Expanding the research landscape will contribute to improved diagnostic and therapeutic strategies and facilitate the development of more effective AI-driven tools explicitly tailored for endometriosis. A major challenge in utilizing machine learning for diagnosing endometriosis through imaging is acquiring well-curated validation datasets.^46^ Moreover, the diverse characteristics of endometriosis, including various subtypes and sites, complicate the creation of reliable machine-learning models for accurately detecting and characterizing the condition.^47^ Endometriosis diagnosis needs clinical and imaging evaluation, and physicians may hesitate to depend solely on automated algorithms without understanding their decision-making.^48^ For future developments, incorporating multimodal data —such as clinical, laboratory, and genetic information — can potentially improve the effectiveness of machine learning models in diagnosing endometriosis.^49^ Research targeting optimal imaging features and machine learning algorithms for diagnosing endometriosis should emphasize accuracy, interpretability, and clinical relevance.^48^ Advanced imaging techniques like radiomics may offer new methods for machine learning-based diagnosis and monitoring of endometriosis.^50^

## CONCLUSION

Endometriosis is a complex, chronic inflammatory disorder causing severe discomfort, potential organ damage, infertility, and persistent pelvic pain, impairing quality of life. Timely recognition and accurate diagnosis are crucial for starting effective therapies and preventing complications. However, challenges persist in optimizing diagnostics. Recent advancements in artificial intelligence, especially in diagnostic imaging, promise to improve accuracy and efficiency. Supervised machine learning, a key subset of AI, is instrumental in extracting meaningful patterns from large datasets. Facilitating the identification of subtle abnormalities that may otherwise go unnoticed by conventional methods. This technology has shown immense potential in supporting diagnostic workflows by improving the interpretability of imaging modalities such as MRI, TVUS, and CT scans. By leveraging AI-driven image post-processing algorithms, radiologists can achieve more precise delineation of pathological features, reducing subjectivity and time required for image interpretation.

The integration of AI into diagnostic imaging techniques for endometriosis has demonstrated significant improvement in sensitivity and specificity, contributing to more reliable and reproducible diagnostic outcomes. Despite these advances, the field remains underexplored, with limited research and insufficient resources dedicated to the application of AI technologies in endometriosis detection. To further refine these innovative approaches, there is a pressing need for additional large-scale studies and interdisciplinary collaborations focusing on AI-based diagnostic frameworks. These efforts will be essential to bridge existing gaps, enhance clinical utility, and ultimately improve patient outcomes in endometriosis care. Despite the promising capabilities of deep learning methods in identifying endometriotic lesions, research underscores challenges resulting from the limited availability of labeled datasets and the diverse imaging techniques utilized across various studies. These factors hinder the reproducibility of findings and constrain the application of machine learning models in different clinical contexts. Although machine learning (ML) holds potential for imaging endometriosis, many existing studies struggle with issues stemming from small sample sizes and inconsistent imaging protocols. These constraints can lead to overfitting and reduced generalizability of the models. Additionally, the complex architecture of ML models, particularly deep neural networks, hinders their adoption in clinical environments due to concerns about their interpretability and difficulty in integrating them into routine practice.
